# Risk factors for falls in older adults in a South African Urban Community

**DOI:** 10.1186/s12877-016-0212-7

**Published:** 2016-02-24

**Authors:** Sebastiana Zimba Kalula, Monica Ferreira, George H. Swingler, Motasim Badri

**Affiliations:** Division of Geriatric Medicine, The Albertina and Walter Sisulu Institute of Ageing in Africa, University of Cape Town, Cape Town, South Africa; International Longevity Centre South Africa, Institute of Ageing in Africa, Department of Medicine, Faculty of Health Sciences, University of Cape Town, Cape Town, South Africa; Department of Paediatrics and Child Health, Faculty of Health Sciences, University of Cape Town, Cape Town, South Africa; Department of Medicine, Faculty of Health Sciences, University of Cape Town, Cape Town, South Africa; King Saud bin Abdulaziz University for Health Sciences, Riyadh, Saudi Arabia

**Keywords:** Falls, Risk factors, Community dwelling, Older people, Low and middle income country

## Abstract

**Background:**

Studies on falls in older adults have mainly been conducted in high income countries. Scant, if any, information exists on risk factors for falls in the older population of sub-Saharan African countries.

**Methods:**

A cross-sectional survey and a 12-month follow-up study were conducted to determine risk factors for falls in a representative multi-ethnic sample of 837 randomly selected ambulant community-dwelling subjects aged ≥65 years in three suburbs of Cape Town, South Africa. Logistic regression models were fitted to determine the association between (1) falls and (2) recurrent falls occurring during follow-up and their potential socio-demographic, self-reported medical conditions and physical assessment predictors.

**Results:**

Prevalence rates of 26.4 % for falls and 11 % for recurrent falls at baseline and 21.9 % for falls and 6.3 % for recurrent falls during follow-up. In both prospective analyses of falls and recurrent falls, history of previous falls, dizziness/vertigo, ethnicity (white or mixed ancestry vs black African) were significant predictors. However, poor cognitive score was a significant predictor in the falls analysis, and marital status (unmarried vs married) and increased time to perform the timed Up and Go test in the recurrent fall analysis but not in both. Other than the timed Up and Go test in recurrent falls analysis, physical assessment test outcomes were not significant predictors of falls.

**Conclusion:**

Our study provides simple criteria based on demographic characteristics, medical and physical assessments to identify older persons at increased risk of falls. History taking remains an important part of medical practice in the determination of a risk of falls in older patients. Physical assessment using tools validated in developed country populations may not produce results needed to predict a risk of falls in a different setting.

## Background

Falls are a major cause of morbidity and mortality in older adults, and the management of falls in these individuals is costly. The incidence and prevalence of falls, and the severity of complications following a fall increase steadily after the age of 60 [1, 2]. In high income countries, unintentional injuries have been listed as the fifth highest cause of death in older adults, after cardiovascular disease, cancer, stroke and pulmonary disorders [3]; the injuries account for 10 % of emergency hospital visits and six per cent of hospital admissions [4]. Falls that do not result in serious injury may still have serious psychological consequences for an older adult, who may fear falling again, and lead to dependence and self-protective immobility.

Falls are often a marker of underlying, preventable problems relating to health and other intrinsic or biological factors, the environment, and behavioural and socio-economic factors. Socio-economic status, determined by low income, low education, a poor housing environment and limited access to social services, is a predisposing factor to the development of chronic disease, which in turn is a risk factor for falls [5, 6]. Unlike biological risk factors for falls, psychosocial risk factors have varied from region to region. Location of residence has been implicated more than ethnicity in the rate of falls in older people [7]. In a study on prevalence of falls in older men in the US, Sweden and Hong Kong, Karlsson et al., 2014, reported that the proportion of falls in Caucasian men was higher in the US than in Sweden, while the proportion of fallers in different ethnic groups within the US was not significantly different. A large primary care study in the United Kingdom reported an inverse relationship between socio-economic status and the incidence of falls [8].

In low and middle income countries, including South Africa, environmental factors may be a greater contributor to intrinsic causes of falls, because of poor infrastructure in numerous geographic areas: In particular, poorly maintained external environments, roads and public buildings; poor or non-existent street lighting; overcrowding and hazards in small dwellings in urban informal settlements; and outdoor hazards where older people access amenities. Assessment of an older adult at risk of a fall, and identification of risk factors that may contribute to a fall as well as circumstances surrounding a fall are important in the design and implementation of intervention programs to prevent falls.

Although substantial knowledge exists on falls in older people and associated risk factors in high income countries, only scant knowledge is available in this subject area in low and middle income countries, particularly in the sub-Saharan Africa region [9, 10]. No falls prevention and education programs are implemented in South Africa. The study reported here sought to identify risk factors for falls in older adults in an urban community in South Africa. The study outcome it was contended, could contribute to knowledge in this area in low and middle income countries as a whole, as well as inform the development of protocols and intervention programs to prevent falls in the countries. Our article focuses on risk factors established for falls in the study population.

## Methods

### Study design

A cross-sectional descriptive/analytic survey with a 12-month follow-up study was conducted in 2009/2010 in three purposively selected suburbs of Cape Town: Plumstead (which has a majority white population), Wynberg Central (with a majority coloured (mixed ancestry) population) and Gugulethu (with a majority black African population). Data were collected using structured questionnaires, and through physical assessments and measurements. The questionnaires, constructed in English, were translated into isiXhosa (the local African language) for administration in the black African sub-sample, back translated and piloted.

Due to a lack of prevalence data on falls in the region, a pilot study was conducted in the study population to calculate the sample size for the study. A fall prevalence of 23.8 % was established in 105 subjects with a mean age of 75.7 years. To allow for comparisons of prevalence rate of falls between study populations from the three suburbs – i.e. three sub-samples, at a level of significance of 5 % and 80 % power, a sample of 280 was required from each suburb, with a total sample of 840.

The study required a portable chair that was easily transportable to use, to test lower limb power and balance of subjects during interviews in the subjects’ dwelling. An equipment validation study was conducted prior to the baseline survey to validate the portable chair that would be used in the study setting [11].

### Setting and participants

The selection of three suburbs of Cape Town for the sampling frame drew on the 2001 population census – the most recent census data available at the time. Each of the suburbs has a comparatively large older population and collectively were representative of South Africa’s multi-ethnic population. The percentage population aged ≥ 65 years of each suburb was as follows: Plumstead: 15 %; Wynberg: 11 %; and Gugulethu: 5.6 % [12]. The economic profile of the suburbs differs: Gugulethu falls in the poorest Human Development Index (HDI) and the Cape Town City Development Index (CDI). The composite index used is derived from attributes such as low income, low educational attainment, high unemployment, jobs in relatively unskilled occupations and the degree of access to a range of services [13].

Inclusion criteria for the study were: Age ≥ 65 years; resident in one of the three suburbs in the sampling frame; able to walk independently, with or without a walking aid; and ability to give informed consent to participate in the study. Approval to conduct the study was obtained from the Ethics and Research Committee of the Faculty of Health Sciences at the University of Cape Town (REC REF: 126/2008). Written consent was obtained from all subjects prior to enrolment in the study.

Information at baseline and at 12-month follow-up was gathered from subjects in their dwelling by specially trained field workers fluent in the home language of the subjects (English or isiXhosa). Study subjects who were recruited into the study at baseline were revisited at approximately 12 months following the baseline survey, at the same address that was recorded at baseline. Informed consent was obtained both at baseline and follow-up prior to the interview and performance of physical assessments. The information gathered pertained to a history of falls in the previous 12 months and the presence of risk factors known to be associated with falls. Physical assessments were performed to record measurements that could identify a propensity to fall. No telephonic interviews or diary recordings were conducted in the follow-up period due to the varied literacy levels of the study sample and limited participants’ access to telephones.

A fall was defined for study participants as an episode in which a person unintentionally comes to rest on the ground, floor or other lower level with or without injury. The definition included falls which resulted from contributing factors, such as syncope, but excluded falls due to a violent blow, an epileptic seizure, or the sudden onset of paralysis such as in a stroke [14, 15].

### Variables recorded

Socio-demographic information included: age, gender, marital status, living arrangement, race (ethnicity), level of education, occupational history, type of housing, main source of income and household socio-economic status (SES). (An SES index comprised the score on an 8-item index of household items in functional order (telephone or cellular phone, stove or burner, electricity, television set, radio, refrigerator, washing machine and car)). Self–reported data were collected on chronic medical conditions diagnosed by a doctor or a nurse (hypertension, stroke, Parkinson’s disease, diabetes, memory loss, depression, arthritis, chronic lung disease, ischaemic heart disease, peripheral vascular disease, heart failure, foot problems, dizziness or vertigo and cancer); bladder function; medications (including over-the-counter (OTC) medications, and herbal and traditional preparations); and alcohol consumption. Cognitive status was assessed using the Short Orientation Memory Concentration Test (SOMCT) [16], also known as the 6 Item Cognitive Impairment Test (6 CIT). A high score signifies cognitive impairment. Mood state was assessed using the short form Geriatric Depression Scale (GDS) [17].

Functional state was evaluated through an assessment of performance of basic activities of daily living (ADLs) [18] and instrumental activities of daily living (IADLs) [19]. Gait, balance and mobility were assessed by observing ability to perform the following manoeuvres: One legged flamingo stand (Unipedal stance) [20]; timed Up and Go test [21]; Five Chair Stands (Sit-to-stand test) [22]; Feet together (Romberg) test; and the Near Tandem Stand test [23, 24]. Mobility was measured using a five-point scale on self-assessed mobility, with or without a walking aid: (independent, get around without walking aid but with difficulty, get a round with a cane, with a frame, with help of another person). Upper limb muscle strength was measured by the handgrip test using a Hydraulic Hand Dynamometer (Jamar 5030 J1) [25]. The average of the highest of three readings for the right hand and the left hand was used for the analysis.

Vision was assessed according to self-reported ability to recognise a face at 4 m (across the road) or to see objects at arm’s length. Hearing was assessed based on self-reported ability to hear a direct conversation and an ability to follow group conversation. Information on subjects’ general health was measured with the use of a five-point self-rated health status scale (very good, good, average, poor, or very poor) and perceived change in general health compared to a year ago (better, about the same, or worse). Overall health status was measured with the use of the Medical Outcomes Study Short Form – 36 (SF-36) health survey [26]. Finally, blood pressure and pulse were checked (supine and erect for 3 min), and weight (in kilograms), height (in metres), and waist and hip circumference (in centimetres) were measured.

### Falls

History of falls and recurrent falls in the past 12 months, the circumstances of the fall/s, events and symptoms associated with the fall/s, and types of injuries sustained and medical attention sought and received were recorded, as well as change in normal daily activities as a result of a fall. In addition, subjects were asked about fear of falling, and to identify indoor and outdoor obstacles in their environment that might precipitate a fall.

### Data management and statistical analysis

All fixed item responses were coded, and open ended responses were categorised and coded. Data codes were entered into a Microsoft Access 2003 database and data were analysed using the SPSS software version 19 (IBM SPSS Inc., Chicago Illinois). Frequency distributions were run to check that all variables were in the acceptable range, and outlying values were checked and corrected as indicated. The prevalence and incidence of falls was calculated with a 95 % confidence interval. For continuous variables, data were tested for normality of distribution using the Shapiro-Wilks test. Parametric tests were used for normally distributed data and non-parametric tests for non-normally distributed data. The relationship between falls and potential predictors of a fall and recurrent falls was determined using a backward stepwise logistic regression analysis with a probability of 0.05 for a variable to enter the model and 0.10 for removal. Strength of association was expressed as an odds ratio with 95 % confidence intervals (CIs). All tests were two-sided and a *p*-value less than 0.05 was considered significant.

## Results

The baseline survey comprised 837 participants. Of these, 366 (43.7 %) resided in Wynberg and 640 (76.5 %) were female. The follow-up study included 632 subjects: 303 (47.9 %) resided in Wynberg and 488 (77.9 %) were female. Two hundred and five (24 %) subjects were lost to follow-up. Socio-demographic characteristics by area of residence (suburb) of the baseline sample are shown in Table 1. The majority of residents in Gugulethu were black African (97.9 %), in Plumstead, white (67.7 %) and in Wynberg, coloured (mixed ancestry) (92.6 %). The number of respondents in skilled and professional/managerial occupations during their working life was higher in Plumstead and Wynberg than in Gugulethu. The majority of respondents in Gugulethu had a primary school level of education (Table 1). The socio-economic status (SES) scores of respondents in both Plumstead and Wynberg, (median and interquartile range (IQR) 8 (7–8)) were significantly higher than that of respondents in Gugulethu 6 (5–6), *p* value <0.001 (Table 2). The number of medical conditions and the number of drugs taken were higher in residents of Plumstead and Wynberg, but respondents in Gugulethu reported a higher frequency of hypertension, diabetes, arthritis and poor memory, and rated their health as poor (Table 2). In general, women reported a higher number of comorbid conditions than men (median, IQR: 3 (2–4) for men and 3 (2–5)) for women, *p* = 0.001) and medication intake (including over-the-counter drugs) (median, IQR: 4 (2–5) for men and 4 (2–6) for women, *P* = 0.021).

**Table 1:** Socio-demographic characteristics of the baseline sub-samples by area of residence (suburb) (*n* = 837)

Characteristic	Gugulethu	Plumstead	Wynberg	*P* value
	*N* = 283	*N* = 188	*N* = 366	
Age, mean (SD) years	74 (6.1)	75 (7.1)	74 (6.2)	0.225
Female (N (%))	231 (81.6)	145 (77.1)	264 (72.1)	0.018
Marital status (N (%))				<0.001
Married	50 (17.7)	68 (36.2)	144 (39.3)	
Widowed	220 (77.7)	95 (50.5)	168 (45.9)	
Divorced/separated	4 (1.4)	10 (5.3)	22 (6.0)	
Never married	9 (3.2)	15 (8.0)	32 (8.7)	
Lives alone (N (%))	4 (1.4)	67 (35.6)	43 (11.7)	<0.001
Residents per household (median (IQR))	7 (5–9)	2 (1–3)	3 (2–4)	<0.001
Ethnic group (N (%))				<0.001
Black African	227 (97.9)	5 (2.7)	1 (0.3)	
Coloured	3 (1.1)	50 (26.6)	339 (92.6)	
Indian	3 (1.1)	2 (1.10)	17 (94.6)	
White	0 (0.0)	131 (69.7)	9 (2.5)	
Level of education (N (%))				<0.001
No formal schooling	24 (8.5)	4 (2.1)	17 (4.6)	
Primary schooling	160 (56.5)	10 (5.3)	71 (19.40)	
Secondary schooling	94 (33.2)	150 (79.8)	255 (69.7)	
Post school qualification	5 (1.8)	24 (12.8)	23 (6.3)	
Occupational category (N (%))				<0.001
Unskilled	224 (79.2)	21 (11.2)	64 (17.5)	
Skilled	55 (19.4)	131 (69.7)	250 (68.3)	
Professional/Managerial	4 (1.4)	36 (19.1)	52 (14.2)	
Receipt of a social pension (yes)	281 (99.3)	90 (47.6)	280 (76.7)	<0.001
SES index (median, IQR)	6 (5–6)	8 (7–8)	8 (7–8)	<0.001

Unless otherwise stated, numbers are n (%). IQR = interquartile range SD = Standard deviation. SES = Socio-economic status. *P*-value: ANOVA test for means, Kruskal-Wallis test for medians, χ^2^ test for categorical variables

**Table 2:** Health and lifestyle characteristics of the baseline samples by area of residence (suburb) (*n* = 837)

Characteristic	Gugulethu	Plumstead	Wynberg	*P* value
	*n* = 283	*n* = 188	*n* = 366	
Self-rated health status (N (%))				<0.001
Very good, Good	54 (19.1)	140 (74.5)	207 (56.6)	
Poor, Very poor	229 (80.9)	48 (25.5)	159 (43.4)	
Perceived health compared to a year ago (N (%))				<0.001
Better/same	269 (95.1)	170 (90.4)	307 (83.9)	
Worse	14 (4.9)	18 (9.6)	59 (16.1)	
Self-rated mobility (N (%))				0.281
Independent	237 (83.7)	163 (86.7)	298 (81.4)	
With difficulty	46 (16.3)	25 (13.3)	68 (18.6)	
Reported previous fall (Yes)	15 (5.3)	74 (39.4)	132 (36.1)	<0.001
Reported previous recurrent falls (Yes)	1 (0.4)	34 (18.1)	57 (15.6)	<0.001
Self-reported medical conditions(N (%))				
Hypertension (Yes)	219 (77.4)	125 (66.5)	227 (62.2)	<0.001
Arthritis (Yes)	178 (63.1)	121 (64.4)	202 (55.2)	0.046
Cardiovascular disease (Yes)	87 (30.7)	96 (51.1)	211 (57.7)	<0.001
Foot problems (Yes)	31 (11.0)	59 (31.4)	127 (34.7)	<0.001
Diabetes (Yes)	146 (51.6)	37 (19.7)	91 (24.9)	<0.001
Dizziness, vertigo (Yes)	17 (6.0)	56 (29.8)	90 (24.7)	<0.001
Chronic lung disease (Yes)	24 (8.5)	26 (13.8)	43 (11.8)	0.174
Depression (Yes)	22 (7.8)	34 (18.1)	32 (8.7)	0.001
Stroke (Yes)	12 (4.2)	17 (9.0)	38 (10.4)	0.014
Poor memory (Yes)	36 (12.7)	4 (2.1)	23 (6.3)	<0.001
Cancer (Yes)	2 (0.7)	21 (11.2)	22 (6.0)	<0.001
Parkinson’s disease (Yes)	7 (2.5)	5 (2.70)	11 (3.0)	0.916
Number of comorbidities (median, IQR)	3 (2–4)	4 (2–5)	3(2–5)	<0.001
Total drug intake (median (IQR)	3 (1–4)	5 (3–7)	5 (3–7)	<0.001
Poor urine control (Yes)	41 (14.5)	49 (26.1)	101 (27.6)	0.001
Impaired hearing (Yes)	72 (25.4)	63 (33.5)	113 (30.9)	0.135
Vision (N (%))				
Far: poor (Yes)	40 (14.1)	8 (4.3)	26 (7.1)	<0.001
Near: poor (Yes)	44 (15.6)	5 (2.7)	10 (2.7)	<0.001
Tobacco use				<0.001
Current (Yes)	9 (3.2)	22 (11.7)	69 (18.9)	
Previous (Yes)	30 (10.6)	70 (37.2)	99 (27.0)	
Alcohol consumption				<0.001
Current (Yes)	6 (2.1)	78 (41.5)	34 (9.3)	
Previous (Yes)	31 (11.0)	15 (8.0)	38 (10.4)	
BMI (median, IQR)	30 (27–34)	27 (23–30)	27 (24–30)	<0.001

For dichotomous variables used Yes or No, Unless otherwise stated, numbers are n (%). IQR = interquartile range, SD = Standard deviation. *P*-value: ANOVA test for means, Kruskal-Wallis test for medians, χ^2^ test for categorical variables

Cardiovascular disease includes angina, heart failure and peripheral vascular disease, lung disease = chronic obstructive airways disease or asthma

Compared to those who were followed up over the 12 months period (*n* = 632), those lost to follow-up (*n* = 205) were older (mean age (SD)) 73.7 (6.2), vs 76.2 (6.7) *p* <0.001), a higher proportion rated their health as poor/ very poor (70.2 % vs 46.2 %, *p* < 0.001) and reported having difficulties with mobility (26.8 % vs 13.3 %, *p* < 0.001). However, there was no difference in the reported history of falls in the previous 12 months (26.9 % vs, 24.4 %, *p* =0.568) or recurrent falls (11.1 vs 10.7, *p* = 0.891).

Prevalence rates of 26.4 % for fall and of 11 % for recurrent falls at baseline and 21.9 % for falls and 6.3 % for recurrent falls were estimated at follow-up. Of the three suburbs, residents of Gugulethu reported the lowest percentage of falls (5.3 %) and recurrent falls (0.4 %), while Plumstead residents reported the highest percentage (39.4 %) and recurrent falls (18.1 %). The incidence rate for falls was 8.75 per 1000 person years for Gugulethu, 24.62 per 1000 person years for Wynberg and 62.35 person years for Plumstead. The overall incidence for falls was 29.5 per 1000 person years.

### Risk factors for falls

Health, life style characteristics and physical measurements of fallers and non-fallers of the follow-up sample (n = 632) are shown in Table 3. Fallers had significantly higher self-reported cardiovascular disease, foot problems, dizziness, history of a previous fall and poor urine control. In addition, fallers reported a higher number of medical conditions and took more medications than non-fallers. The median hand grip measurement was lower in fallers than non-faller (Table 3). However, in the stepwise logistic regression analyses at baseline, factors associated with any fall in the previous 12 months were self-rated poor health, perceived worse health than a year ago, self-reported medical conditions (Parkinson’s disease, poor urine control), greater number of co-morbid conditions, the number of medications taken, a high cognitive score on the Short Orientation Memory Concentration test (SOMCT) suggesting cognitive impairment, occupational history and the number of household residents. Predictors of recurrent falls were perceived worse health than a year ago, number of medications, the number of co-residents , and medical conditions (previous stroke, self-reported Parkinson’s disease, foot disorders and poor urine control) (Table 4).

**Table 3:** Health and lifestyle characteristics according to fall history in the follow-up sample (*n* = 632)

Characteristic	Fallers	Non-Fallers	*P* value
	*n* = 170	*n* = 462	
Self-rated health status (N (%))			0.040
Very good, Good	105 (61.8)	243 (52.6)	
Poor, Very poor	65 (38.2)	219 (47.4)	
Perceived health compared to a year ago (N (%))			0.045
Better/same	147 (86.5)	424 (91.8)	
Worse	23 (13.5)	38 (8.2)	
Self-rated mobility (N (%))			0.617
Independent	142 (83.5)	378 (81.8)	
With difficulty	28 (16.5)	84 (18.2)	
Self-reported previous fall (yes)	64 (37.6)	75 (16.2)	<0.001
Self-reported previous recurrent falls (yes)	34 (18.1)	57 (15.6)	<0.001
Self-reported medical conditions			
Hypertension (Yes)	112 (65.9)	326 (70.6)	0.258
Arthritis (Yes)	109 (64.1)	278 (60.2)	0.367
Cardiovascular disease (Yes)	95 (55.9)	159 (34.4)	<0.001
Foot problems(Yes)	69 (40.6)	108 (23.4)	<0.001
Diabetes (Yes)	50 (29.4)	143 (31.0)	0.709
Dizziness, vertigo (Yes)	51 (30.0)	77 (16.7)	<0.001
Chronic lung disease (Yes)	13 (7.6)	36 (7.8)	0.952
Depression (Yes)	12 (7.1)	27 (5.8)	0.574
Stroke (Yes)	15 (8.8)	22 (4.8)	0.054
Poor memory (Yes)	6 (3.5)	13 (2.8)	0.640
Cancer (Yes)	16 (9.4)	27 (5.8)	<0.001
Parkinson’s disease (Yes)	7 (4.1)	6 (1.3)	0.027
Number of medical conditions (median, IQR)	4 (2–5)	3(2–4)	<0.001
Number of medications (median (IQR)	5 (3–7)	4 (2–5)	<0.001
SES index (median, IQR)	8 (7–8)	7(6–8)	<0.001
Residents per household (median (IQR))	3 (2–4)	3 (2–5)	0.002
Hand grip (kg) (median IQR)	9.5 (2–16)	10 (6–16)	0.044
Timed Up and Go test (seconds)	14 (11–19)	14 (11–20)	0.538
Chair stands (seconds)	14 (10–17)	15 (11–20)	0.004
BMI (median, IQR)	27 (24–32)	28 (25–32)	<0.001
Poor urine control (Yes)	62 (36.5)	97 (21.0)	<0.001
Impaired hearing (Yes)	58 (34.1)	94 (20.3)	<0.001
Vision (N (%))			
Far: poor (Yes)	8 (4.7)	35 (7.6)	0.204
Near: poor (Yes)	1 (0.6)	32 (6.9)	0.001
Tobacco use (N (%))			0.001
Current (Yes)	23 (13.5)	56 (12.1)	
Previous (Yes)	58 (34.1)	93 (20.1)	
Alcohol consumption (N (%))			0.090
Current (Yes)	33 (19.4)	64 (13.9)	
Previous (Yes)	16 (9.4)	31 (6.7)	

Unless otherwise stated, numbers are n(%). IQR = interquartile range. *P*-value: ANOVA test for means, Kruskal-Wallis test for medians, χ^2^ test for categorical variables

Cardiovascular disease includes angina, heart failure and peripheral vascular disease, lung disease = chronic obstructive airways disease or asthma

**Table 4:** Risk factors for falls: logistics regression analysis, baseline survey, non-fallers versus fallers and non-fallers versus recurrent fallers

Factor	Non fallers versus fallers				Non fallers versus Recurrent fallers				
	OR	95 % CI		*P*-value	Factor	OR	95 % CI		*P*-value
Residents per household	0.90	0.83	0.97	0.014	Residents per household	0.88	0.78	0.99	0.038
Number of drugs	1.16	1.08	1.24	<0.001	Number of drugs	1.22	1.11	1.34	<0.001
Number of medical conditions	1.19	1.05	1.36	0.006	Self-reported previous stroke				
Cognitive score (SOMCT)	1.04	1.01	1.08	0.006	No	1			
Self-rated health					Yes	3.36	1.69	6.80	0.001
Very good/good	1				Perceived worse health than a year ago				
Poor/very poor	1.60	1.09	2.34	0.016	Better/Same	1			
Perceived worse health than a year ago					Worse	2.10	1.09	4.06	0.027
Better/Same	1				Self-reported Parkinson’s disease				
Worse	2.31	1.30	4.13	0.005	No	1			
Self-reported Parkinson’s disease					Yes	5.84	1.90	17.88	0.002
No	1				Self-reported poor urine control				
Yes	3.29	1.21	8.97	0.020	No	1			
Self-reported poor urine control					Yes	1.96	1.15	3.35	0.014
No	1				Self-reported foot disorder				
Yes	1.67	1.13	2.67	0.010	No	1			
Occupation					Yes	2.26	1.33	3.83	0.002
Unskilled	1								
Skilled	2.21	1.40	3.50	0.001					
Managerial/Professional	2.73	1.46	5.12	0.002					

*SOMCT* Short Orientation Memory Concentration Test, *SES* Socio-economic status, *IQR* interquartile range, *OR* odds ratio, *CI* confidence interval. *P*-value: Wald test

On Stepwise logistic regression analysis using risk factors for falls reported at baseline and a fall/s reported prospectively, predictors of any fall and recurrent falls were: history of a previous fall, dizziness or vertigo, ethnicity (being white or of mixed ancestry), marital status (not being married), and poor (increased) cognitive score on the Short Orientation Memory Concentration Test (SOMCT). In addition, increased time to complete the timed Up and Go test was a predictor of recurrent falls (Table 5). The risk of a fall increased two-fold and that of recurrent falls was eight-fold in those who had history of a previous fall. For every second increase in time to complete the timed Up and Go test, the risk of recurrent falls increased by six per cent. For each point increase on the SOMCT score, the risk of a fall increased by four per cent (Table 5).

**Table 5:** Risk factors for falls and recurrent falls: stepwise logistics regression analysis, a fall in the follow up period and risk factors reported at baseline, non-fallers versus fallers and non-fallers versus recurrent fallers

	Non fallers versus Fallers				Non fallers versus Recurrent Fallers				
Factor	OR	95 % CI		*P*-value	Factor	OR	95 % CI		*P*-value
History of previous fall					History of previous fall				
No	1				No	1			
Yes	2.28	1.49	3.48	<0.0001	Yes	8.66	3.69	20.29	<0.0001
Dizziness /vertigo					Dizziness /vertigo				
No	1				No	1			
Yes	1.87	1.19	2.93	0.007	Yes	2.46	1.14	5.33	0.022
Ethnic group					Ethnic group				
Black African	1				Black African	1			
Mixed ancestry	1.98	1.11	3.51	0.020	Mixed ancestry	5.19	1.28	21.15	0.021
White	3.32	1.65	6.69	0.001	White	7.04	1.39	35.68	0.018
Cognitive score (SOMCT)	1.04	1.01	1.08	0.021	Cognitive score (SOMCT)	1.06	.99	1.14	0.068
					Marital status				
					Married	1			
					Not married	2.87	1.16	7.11	0.023
					Timed Up and Go test (secs)	1.06	1.02	1.10	0.002

*SOMCT* Short orientation Memory Concentration Test score (measure on a continuous scale), *Secs* seconds, *OR* odds ratio, *CI* confidence interval. *P*-value: Wald test

## Discussion

Factors associated with falls at baseline in an urban community-dwelling older population of Cape Town were identified as being, poor cognitive function, self-reported medical conditions (Parkinson’s disease, stroke, foot disorders and urinary incontinence), number of comorbid conditions, number of medications, self-rated health as poor, perceived worse health than a year ago, number of residents in a household, and occupational history (skilled or managerial/professional). However, risk factors that predicted falls prospectively were: history of a previous fall, dizziness and vertigo, ethnicity (being white or of mixed ancestry, marital status (not being married), and increase time taken to perform the timed Up and Go test and poor cognitive score. To our knowledge, our study is the first to be conducted on falls in older adults in South Africa’s multi-ethnic and socio-economically diverse population.

The SES index of subjects in Gugulethu who were largely black Africans was low; the majority had a primary school education and had been unskilled workers. The widely different occupational history and socio-economic status of the subjects in the three suburbs may have influenced propensity to fall. The findings are thus in contrast with those of studies that have shown an increased risk of falls in individuals with low socio-economic status [8].

Unexpected findings in this study were a lack of significant association between age or gender, and a fall or recurrent falls. Advanced age has been associated with falls due to a combination of physiological factors that predispose to changes in gait and/or balance attributed to decreased muscle strength and an increase in co-morbid conditions associated with age [27, 28]. Although gender was not a predictor of falls, similar to findings in other studies [6, 29, 30], women had a higher number of comorbid conditions and used more drugs than men, and reported a higher number of recurrent falls. Women may indeed be at high risk of falls because of a higher number of comorbid conditions and associated high usage of medications, and gait and/or balance disorders [31].

At baseline, poor cognitive status was associated with falls. Poor cognitive status (impaired motor planning and focussing attention) and impaired activities of daily living [32] increase the propensity to fall. In addition, adverse effects of medications to treat depression and other behavioural and psychological disorders associated with cognitive impairment increase a risk of falls in depressed and cognitively impaired older persons [28]. Perceived poor health, a risk factor for falls in this study, has similarly been reported as a risk factor for falls in other studies [32–37]. Self-rated health status has been found to be a reliable and sensitive measure of health status, and to have implications for maintenance of functional ability in daily life and for survival [38]. Physical and mental disability and impairment caused by comorbid conditions contribute to a sense of poor general health, and impairments predispose to an increased risk of a fall. Chronic medical conditions (Parkinson’s disease and stroke) were independently associated with falls. These medical conditions affect muscle strength, gait and balance, and in some cases are associated with the development of cognitive impairment. An additional risk factor for falls in such individuals is a predisposition to adverse effects of medication such as those used to control symptoms of Parkinson’s disease (postural hypotension, choreiform, dystonic and dyskinetic movements, confusion), or to control risk factors for stroke (antihypertensive).

Self-reported poor urine control was associated with risk for falls at baseline. Urinary incontinence, urge incontinence in particular, has been associated with falls in community-dwelling older people [39, 40]. An association between urinary incontinence and risk of falls could be due to confounding by co-morbidities (e.g. stroke or acute illness as well as due to hurrying to reach a toilet, increasing the likelihood of a trip or slip). Self-reported foot disorders were associated with recurrent falls at baseline. The effects of foot disorders such as corns, hallux valgus and pain alter gait and balance, and have been reported to increase the risk of falling [41, 42].

Total number of medications taken, unlike in other studies [33, 43–46], was independently associated with falls in the baseline sample. This finding is similar to findings of a study by Perracini and colleagues in Brazil [36], and certain studies in high income countries [33, 47]. An association between total number of medications and fall risk will depend on the prescribing habits of health practitioners (type of medications commonly taken by the subjects), the site of action of the drug and adverse effects of the drugs. The variety of classes of medications prescribed, which results in insufficient numbers of individuals taking a particular class of drug, hampers meaningful contribution to the analyses of the individual drug classes.

In the baseline stepwise logistic regression analyses, living arrangement (number of residents in a household) was independently associated with falls. Having co-residents in a dwelling, common in Gugulethu, was a protective factor from falls. Living arrangement in relation to falls has yielded conflicting findings, ranging from no association [35, 37, 48–52], to a high risk of falls [29, 31–35, 48, 53–55], and being protective [56] in those who live alone. Living alone may predispose individuals to depression, poor health and loneliness, and/or increase the likelihood of their carrying out risky physical activities that may predispose them to a fall. Level of mobility was not significantly different in the three sub-samples, yet Gugulethu residents reported far fewer falls than residents of Plumstead and Wynberg. It may be speculated that Gugulethu residents with general low social economic status, and mostly engaged in unskilled labour, had probably engaged in physically demanding occupations such as manual labour or agriculture. This type of occupational engagement may have given them an advantage in muscle reserve capacity and function, and better maintenance of gait and balance.

In the prospective sample, history of a previous fall was a significant predictor of falls ((OR = 2.33; 95 % CI 1.52–3.56) for a fall and (OR = 10.49; 95 % CI 4.55–24.17) for recurrent falls)). History of a previous fall is not a risk factor *per se* [57], but should alert a health care professional to investigate and manage physiological deficits and chronic medical conditions, and for an older adult to take appropriate steps to change behaviour to prevent more falls.

As with history of a previous fall, ethnic group may not be a risk factor for falls *per se*, but a proxy for multiple interacting physical, mental, cultural and life style factors that collectively contribute to a fall in the presence of relevant environmental precipitants. Ethnicity as a risk factor for falls has yielded conflicting results. Most studies on ethnicity and falls have conducted in the US and show no significant difference in fall rates between Caucasians and African Americans [58, 59]. In the study by Hanlon and colleagues (2002), being white was a risk factor for any fall but there was no significant difference between whites and African Americans in the risk to recurrent falls. Self-report dizziness/vertigo was an independent predictor of falls at follow-up. Dizziness has multifactorial causation: Other studies have reported dizziness as a risk factor for falls and it has been recognised as a possible geriatric syndrome [60, 61]. An assessment of cardiovascular, depressive and anxiety symptoms, sensory, balance and gait impairments, blood pressure changes and medication review are required to establish the cause of dizziness [60, 61]. Of the physical measurements, only the timed Up and Go test was a predictor of recurrent falls in the follow-up survey. The timed Up and Go test correlates with functional capacity, a decrement of which increases a propensity to fall [21].

Although a number of physical assessment tests were performed, the results were generally not predictors of falls in subjects. Apart from the Short Orientation Memory Concentration test score and the timed Up and Go test, most physical measurements and tests of balance or physical strength (vital signs, hand grip strength, five chair stands, feet together, semi tandem stand) used in the study were not independently associated with a fall. Conversely, difficulty in performing the sit-to-stand chair test was not a predictor of falls. The chair sit-to-stand test tests several functional components, including muscle strength, balance, and co-ordination, a joint range of motion and exercise tolerance, and psychological status [62]. Difficulty in performing the sit-to-stand chair test was particularly evident in Gugulethu residents, who reported the lowest number of falls. Cultural interpretations, attitudes and psychological factors may have played a role when subjects were asked to perform certain timed tests, which had a bearing on the poor correlation between performance in physical tests and the risk of falls in some subjects.

The poor association with physical measurements demonstrated supports the finding and conclusion of Tiedemann and colleagues (2008): Tests demonstrate poor to fair sensitivity and specificity in identifying older people at risk of multiple falls. The authors recommended that physical assessment tests be used only to identify those in need of full evaluation; individually, the tests are not predictive of persons at risk of falls as such [63].

Strengths and limitations of the study are as follow: The cross-sectional study design with a 12-month follow-up provided baseline information on participants. However, determination of risk factors and their association with falls was hindered by the cross-sectional data collection and retrospective recall of falls both at baseline and follow-up. A temporality between a fall and a risk factor could not be established within the cross-sectional design. The exclusion of subjects who were unable to walk or to give consent to participate in the study may have excluded individuals with physical and mental frailty who are at increased risk of falls, and led to an underestimate of the prevalence of falls in this community. Subjects lost to follow-up, may have differed from those who remained in the study and the outcome is likely to be an under-estimate of the actual incidence, although the report of falls prior to baseline was not significantly different between those lost to follow-up and those who remained in the study. For rare outcomes such as recurrent falls, and risk factors such as drug classes, a larger sample would be required to allow for adequate analyses.

## Conclusion

Findings of prospective stepwise logistic regression analyses of the study data showed the main independent risk factors for falls to be history of previous falls and ethnicity. Self-reported conditions, and not physical assessment outcomes, were generally associated with risk of a fall. Despite technological advances in medical practice, history taking remains an important part of the determination of a risk of falls in older patients. Hence, particular attention should be paid to history taking supplemented by physical assessment outcomes in predicting fall risk. Physical assessment performed with tools validated in developed country populations may not produce the results predicting of fall risk in developing country settings. Future studies are indicated to determine differential fall risk in the multi-ethnic South African population.

### Availability of supporting data

Data are accessible on figshare: https://figshare.com/account/login.
